# Endoscopic ultrasound-guided hepaticojejunostomy for complete biliary anastomotic stricture: the echo-free space technique for scope insertion in surgically altered anatomy

**DOI:** 10.1055/a-2368-3932

**Published:** 2024-08-07

**Authors:** Michihito Kono, Shunsuke Omoto, Mamoru Takenaka, Akito Furuta, Shunsuke Ogata, Taro Inoue, Wataru Ono

**Affiliations:** 1 13737 Gastroenterology, Kishiwada Tokushukai Hospital, Osaka, Japan; 2 145700 Gastroenterology, Kobe Tokushukai Hospital, Kobe, Japan; 3 326473 Gastroenterology and Hepatology, Kindai University Hospital, Osaka-Sayama, Japan

Postoperative biliary strictures are estimated to occur in 2.6% of patients. When endoscopic treatment is difficult, they can be treated with endoscopic ultrasound-guided hepaticojejunostomy (EUS-HJS) using a forward-viewing linear endoscope 1 2 3 4 . However, in many institutions, the forward-viewing scope is not readily available, making immediate intervention difficult. We have developed a safe and reliable method for inserting a side-viewing linear endoscope using the “echo-free space” technique 5 .

We present the case of a 71-year-old man who underwent total pancreatectomy and choledochojejunostomy for pancreatic cancer. After 8 months, he developed cholangitis due to an anastomotic stricture and was referred to our department. Single-balloon endoscopic retrograde cholangiopancreatography (ERCP) and EUS-guided hepaticogastrostomy (EUS-HGS) were attempted, but the patient continued to have recurrent cholangitis. We therefore decided to perform EUS-HJS from the anastomotic site.

The single-balloon enteroscope was first inserted up to the anastomosis, which was marked with a clip; a 6-Fr endoscopic nasobiliary drainage (ENBD) catheter was placed near the anastomotic stricture ( Fig. 1 ). The scope was switched to a side-viewing linear endoscope (GF-UCT260) and the Braun anastomosis was identified on careful observation of the echo image, with the ENBD catheter used as a guide to reach the HJS ( Fig. 2 ; Video 1 ). The bile duct was punctured through the anastomosis with an 19G EZ Shot 3 Plus (Olympus) and a guidewire was placed ( Fig. 3 ). The stenosis was dilated with a spiral drill dilator (Tornus ES; Olympus) and then with a tapered-tip balloon catheter (REN; Kaneka) up to 4 mm ( Fig. 4 ), and the procedure was completed with the placement of a 7-Fr, 9-cm inside stent ( Fig. 5 ).

**Fig. 1:**
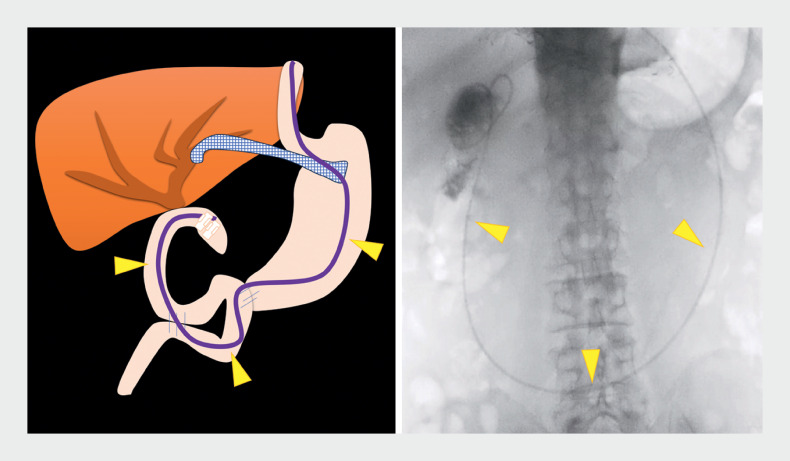
Schematic diagram and fluoroscopic image showing a 6-Fr endoscopic nasobiliary drainage catheter (arrowheads) placed near the anastomotic stricture after a single-balloon enteroscope had been inserted up to the anastomosis, which was marked with a clip.

**Fig. 2:**
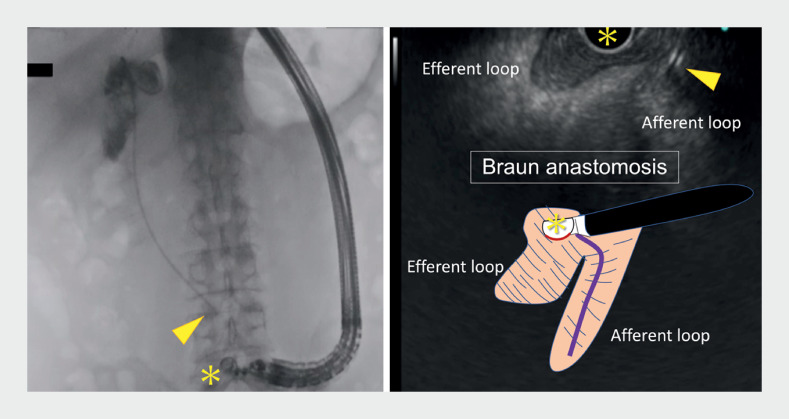
A side-viewing linear endoscope (asterisk) is used to identify the Braun anastomosis, relying on careful observation of the echo image, the endoscopic nasobiliary drainage catheter (arrowhead) is used as a guide to reach the choledochojejunostomy.

Endoscopic ultrasound-guided hepaticojejunostomy is performed in a patient with complete biliary anastomotic stricture using the echo-free space technique to insert the scope into the choledochojejunostomy site.Video 1

**Fig. 3:**
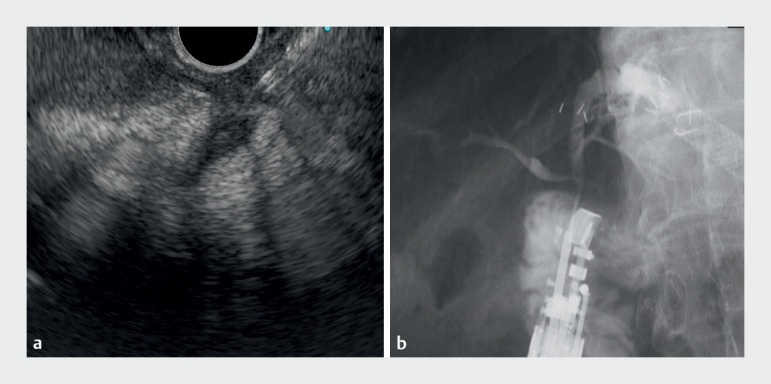
Endoscopic ultrasound and fluoroscopic images showing: **a** the bile duct being punctured through the anastomosis using a 19G needle; **b** the appearance after the injection of contrast medium.

**Fig. 4:**
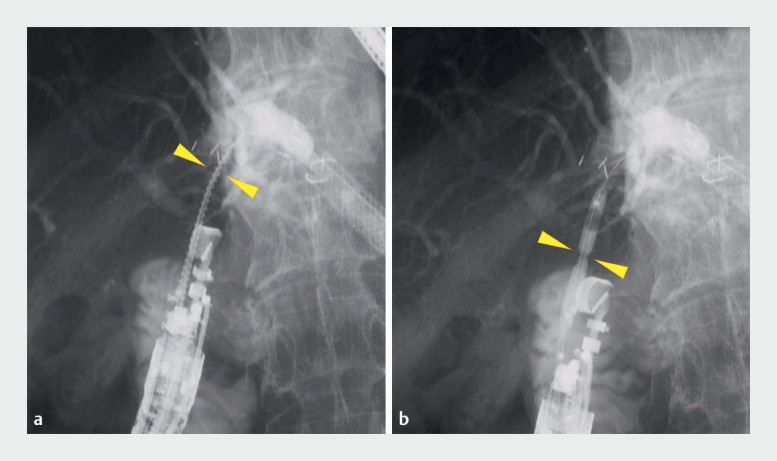
Fluoroscopic images showing the stenosis being dilated with a spiral drill dilator and tapered-tip balloon catheter.

**Fig. 5:**
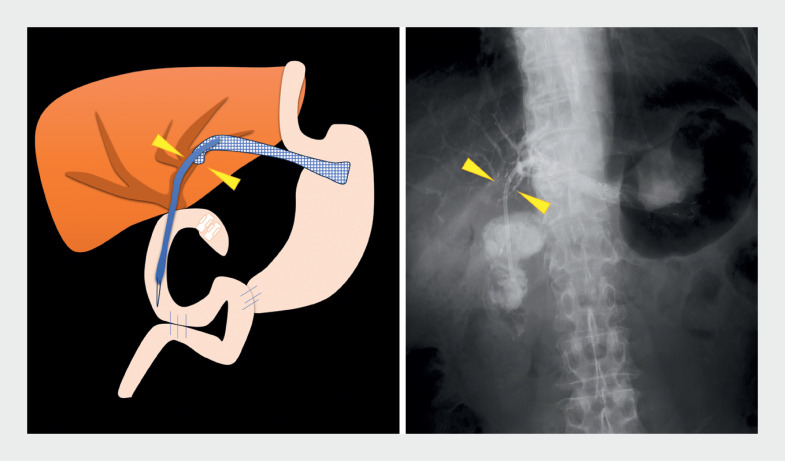
Schematic diagram and fluoroscopic image showing a 7-Fr, 9-cm inside stent (arrowhead) placed to complete the procedure.

This case suggests that the echo-free space technique using a side-viewing linear endoscope can be useful in postoperative patients and represents a new option for EUS-HJS in the treatment of complete biliary anastomotic stricture.

Endoscopy_UCTN_Code_TTT_1AS_2AH

## Notes

Endoscopy E-Videos https://eref.thieme.de/e-videos
*E-Videos* is an open access online section of the journal *Endoscopy* , reporting on interesting cases and new techniques in gastroenterological endoscopy. All papers include a high-quality video and are published with a Creative Commons CC-BY license. Endoscopy E-Videos qualify for HINARI discounts and waivers and eligibility is automatically checked during the submission process. We grant 100% waivers to articles whose corresponding authors are based in Group A countries and 50% waivers to those who are based in Group B countries as classified by Research4Life (see: https://www.research4life.org/access/eligibility/ ). This section has its own submission website at https://mc.manuscriptcentral.com/e-videos .
